# Estimation of ^226^Ra and ^228^Ra Content Using Various Types of Sorbents and Their Distribution in the Surface Layer of the Black Sea

**DOI:** 10.3390/ma16051935

**Published:** 2023-02-26

**Authors:** Ol’ga N. Kozlovskaia, Iuliia G. Shibetskaia, Nikolay A. Bezhin, Ivan G. Tananaev

**Affiliations:** 1 Department of Biogeochemistry, Marine Hydrophysical Institute, Russian Academy of Sciences (MHI RAS), Kapitanskaya Str., 2, 299011 Sevastopol, Russia; 2 Department of Chemistry and Chemical Engineering, Sevastopol State University, Universitetskaya Str., 33, 299053 Sevastopol, Russia; 3 Radiochemistry Laboratory, Vernadsky Institute of Geochemistry and Analytical Chemistry, Russian Academy of Sciences (GEOKHI RAS), Kosygin St., 19, 119991 Moscow, Russia; 4 Department of Nuclear Technology, Far Eastern Federal University, Sukhanov Str., 8, 690091 Vladivostok, Russia

**Keywords:** ^226^Ra, ^228^Ra, seawater, sorbents, sorption, PAN-MnO_2_, nutrients

## Abstract

Radium isotopes have traditionally been used as tracers of surface and underground fresh waters in land–ocean interactions. The concentration of these isotopes is most effective on sorbents containing mixed oxides of manganese. During the 116 RV Professor Vodyanitsky cruise (22 April–17 May 2021), a study about the possibility and efficiency of ^226^Ra and ^228^Ra recovery from seawater using various types of sorbents was conducted. The influence of seawater flow rate on the sorption of ^226^Ra and ^228^Ra isotopes was estimated. It was indicated that the Modix, DMM, PAN-MnO_2_, and CRM-Sr sorbents show the best sorption efficiency at a flow rate of 4–8 column volumes per minute. Additionally, the distribution of biogenic elements (dissolved inorganic phosphorus (DIP), silicic acid, and the sum of nitrates and nitrites), salinity, and ^226^Ra and ^228^Ra isotopes was studied in the surface layer of the Black Sea in April–May 2021. Correlation dependencies between the concentration of long-lived radium isotopes and salinity are defined for various areas of the Black Sea. Two processes control the dependence of radium isotope concentration on salinity: conservative mixing of riverine and marine end members and desorption of long-lived radium isotopes when river particulate matter meets saline seawater. Despite the high long-lived radium isotope concentration in freshwater in comparison with that in seawater, their content near the Caucasus shore is lower mainly because riverine waters meet with a great open seawater body with a low content of these radionuclides, and radium desorption processes take place in an offshore area. The ^228^Ra/^226^Ra ratio derived from our data displays freshwater inflow spreading over not only the coastal region, but also the deep-sea region. The lowered concentration of the main biogenic elements corresponds to high-temperature fields because of their intensive uptake by phytoplankton. Therefore, nutrients coupled with long-lived radium isotopes trace the hydrological and biogeochemical peculiarities of the studied region.

## 1. Introduction

Radium isotopes ^223^Ra, ^224^Ra, ^226^Ra, and ^228^Ra are widely used in oceanology and marine radiochemistry when studying the interaction between seawater and groundwater in the coastal region [1]. Radium isotopes behave conservatively in the marine aquatic system, not being involved in biogeochemical cycles; that is, their supply and consumption are not determined by chemical and biological processes. In addition, the difference in the content of radium isotopes in salt and fresh water is significant (by 5–10 times), which makes it possible to detect the point sources of fresh water reaching the seas and oceans.

Elevated values of radium isotope activity are observed at places of freshwater inflow: estuaries, domestic water discharge points, and sources of submarine groundwater discharge (SGD). Thus, the quantity of radium isotopes, as well as other solutes supplied by surface and underground waters, is closely related to water salinity and can be used to determine the content of solutes in various types of waters entering the marine area [2,3].

Sorption materials of various types find numerous applications for the concentration of radionuclides [4,5,6]. For the recovery of radium isotopes from seawater, sorbents based on mixed oxides of manganese, which is usually described by the simplified formula MnO_2_, are the most widely used. However, it has been found [7,8] that they contain K_1.33_Mn_8_O_16_ and K_1.39_Mn_3_O_6_. They are responsible for the chemisorption of several radionuclides that exist in water in ionic form, for example, radium isotopes.

Acrylate [9,10] and cellulose fibers [10], membrane filters [11], and polypropylene cartridges [12] are widely used as supports for MnO_2_ impregnation. Moreover, it is possible to use granulated MnO_2_ without any support [13].

The sorbent based on acrylic fiber and MnO_2_ obtained by Moore in the 1970s was most widely used for the recovery of radium isotopes. At present, this sorbent is supplied together with radium delayed coincidence counter (RaDeCC) setups designed to measure short-lived ^223^Ra and ^224^Ra isotopes [14] by counting the decay of radon daughter isotopes ^219^Rn and ^220^Rn with a detector. Long-lived ^226^Ra and ^228^Ra isotopes can be measured after radiochemical preparation [15].

In [16], MnO_2_-coated polyamide discs were obtained for the determination of ^224^Ra, ^226^Ra, and ^228^Ra in natural water. This method allows the extraction of radium isotopes from small-volume samples. However, it cannot be used for seawater samples, since at a Ca^2+^ concentration in a solution of more than 2.5 mmol L^−1^, the sorption efficiency of radium isotopes by MnO_2_-coated polyamide discs is less than 50%, and the concentration of Ca^2+^ in the water of various seas and oceans is in the range of 5.5–10 mmol L^−1^. Consequently, the sorption efficiency of radium isotopes by MnO_2_-coated polyamide discs will decrease even more. In addition, to determine ^228^Ra, MnO_2_-coated polyamide discs must be kept for about 6–12 months.

Several methods for the recovery and determination of radium isotopes in natural and biological samples are discussed by the authors of [17]. However, the proposed methods either have a detection limit higher than the concentration of radium isotopes in seawater (30–160 dpm m^−3^), are not intended for extraction from seawater, or show a low recovery efficiency. For example, the sorption efficiency of ^228^Ra 1 g of the commercial sorbent MN resin [13] from Triskem International from 1 L of artificial seawater with a rate of 20 mL min^−1^ is 91.3%. With an increase in the flow rate of artificial seawater to 50 mL min^−1^, the sorption efficiency of ^228^Ra falls to 70%, at a rate of more than 100 mL min^−1^ up to 45%, and more than 200 mL min^−1^ up to 30%, which significantly complicates the processing of a large volume of samples.

At the same time, in our previous work [7], a method for obtaining a sorbent based on PAN fiber and MnO_2_ (PAN-MnO_2_) with better sorption properties was presented. Therefore, the sorption efficiency of ^228^Ra recovery from 50 L of seawater by 1 g of PAN-MnO_2_ sorbent in the range of flow rates of 50–300 mL min^−1^ practically does not change and amounts to 97.4–100%.

It was also shown in our previous work [7] that sorbents of this type allow a simple γ-spectrometric determination of ^7^Be, ^226^Ra, ^228^Ra, and ^234^Th. Using these sorbents for the preconcentration of stable elements is not relevant, since there are already sufficiently sensitive methods for their analysis.

A detailed comparison of various sorption materials used for the recovery of radium isotopes from seawater with summary tables was presented in our previous review paper [18].

There is a lack of data on the concentration of ^228^Ra in the Black Sea. The data presented in [19] for the southwestern part were acquired by interpolation from a single station in the Sea of Marmara. There is also a single measurement from the 1960s of the last century [20] and our recent work [7]. There is also no data on the concentration of short-lived ^223^Ra and ^224^Ra in the Black Sea. 

There is also little information about ^226^Ra concentration in the deep waters of the Black Sea. However, the data are ambiguous. The values of the ^226^Ra concentration in the surface layer obtained in the 1960s (185–246 dpm m^−3^) [20,21] and 1990s (50–88 dpm m^−3^) [22,23,24] differ by a factor of three. The authors link this to an increase in primary production due to industrial and agricultural runoff, and to a lesser extent, a decrease in fluvial sediment loads, owing to extensive river impoundment. However, it seems that in fact, the methods used in the 1960s contained methodological errors, because [21] represents that the concentration of ^226^Ra on the surface and 2000 m deep is the same, which does not reflect the real data. Recent works [23] have shown that the concentration of ^226^Ra on the surface and in the deep part of the Black Sea differs by 2–3 times. Additionally, the concentration of ^226^Ra in river water differs by about 3 times in early and recent works. Therefore, it may seem that differences between old and newer data reflect not real concentration differences, but faulty analytical procedures. At the same time, the authors of [22,23,24] note the interannual variability of the ^226^Ra concentration in the surface layer of the Black Sea. This fact deserves attention, since the concentration was determined by one method using MnO_2_-coated fiber, and the quantity of stations performed is quite representative.

It should be noted that, according to [25,26], the concentration of ^226^Ra increases with depth, while ^228^Ra decreases; this is because of the different half-lives of these isotopes and their ratio in their sources. Therefore, their ratio and their spatial variation are important characteristics for assessing terrestrial freshwater inflow into the ocean. In addition, colloids containing manganese and iron, on which radium isotopes are adsorbed, play an important role in the vertical transfer of radium isotopes, as does the occurrence of redox processes in the suboxic zone. Thus, the factors that determine the seasonal and interannual variability of the ^226^Ra concentration in the Black Sea have not been studied enough, there are practically no data on the ^228^Ra concentration in the Black Sea, and the contribution of various sources of radium isotope input to the Black Sea has not been studied.

This paper is aimed to present the results of a comparison of PAN-MnO_2_ sorbent sorption efficiency we obtained earlier with other commercially available sorbents that have not previously been used for the recovery of radium isotopes from large volumes of seawater, to select the best sorbents for the concentration of ^226^Ra and ^228^Ra from seawater and analyze the content distribution of these isotopes in the surface layer of the Black Sea for assessing terrestrial freshwater inflow.

## 2. Materials and Methods

### 2.1. Sorbents

The characteristics of various types of sorbents based on manganese oxide (Modix (SPE Eksorb Ltd, Yekaterinburg, Russia), MDM and DMM (IPCE RAS, Moscow, Russia), PAN-MnO_2_), phosphorus oxide PD (IPCE RAS, Moscow, Russia), carbonate-containing zirconium dioxide Termoxid 3K (JSC “Inorganic sorbents” Co., Zarechny, Russia), barium silicate CRM-Sr (Institute of Chemistry FEB RAS, Vladivostok, Russia), used to extract ^226^Ra and ^228^Ra from seawater are detailed in [27].

We previously [27,28] investigated these sorbents under static and dynamic conditions for the strontium recovery (as an electronic analog of radium) from seawater. The distribution coefficients and exchange capacities were determined, followed by the construction of output [27] and sorption kinetic curves and sorption isotherms [28].

### 2.2. Hydrological Survey

The distributions of the main hydrological parameters (temperature and salinity) during the RV Professor Vodyanitsky cruise were measured using the IDRONAUT Ocean Seven 320 plus CTD oceanographic probe (Idronaut S.R.L., Brugherio, Italy) with a sensor sampling rate of up to 40 Hz. Measurement errors are as follows: temperature—0.001 °C, electrical conductivity—0.001 mS cm^−1^, and pressure—0.01%.

### 2.3. Sampling and Recovery of ^226^Ra and ^228^Ra

The sampling of seawater during the 116 RV Professor Vodyanitsky cruise (22 April–17 May 2021) to determine the content of ^226^Ra and ^228^Ra was carried out using a submersible pump Unipump Bavlenets BV 0.12-40-U5 (Subline Service LLC, Moscow, Russia). Seawater samples were taken from the surface (3 m below). Water was pumped into plastic containers on board, each with a volume of 250 L, simultaneously filtering seawater through a polypropylene filter with a pore diameter of 0.5–1 μm FCPS1M series (Aquafilter Europe Ltd., Lodz, Poland). Next, seawater was passed through a system of two columns filled with 25 mL of the sorbent each (PAN-MnO_2_, Modix, MDM, DMM, PD, Termoxid 3K, CRM-Sr) at a rate of 2–12 CV min^−1^ (column volumes per minute). Then the sorbents were dried in the air and packaged for analysis in a coastal laboratory.

**Figure 1:**
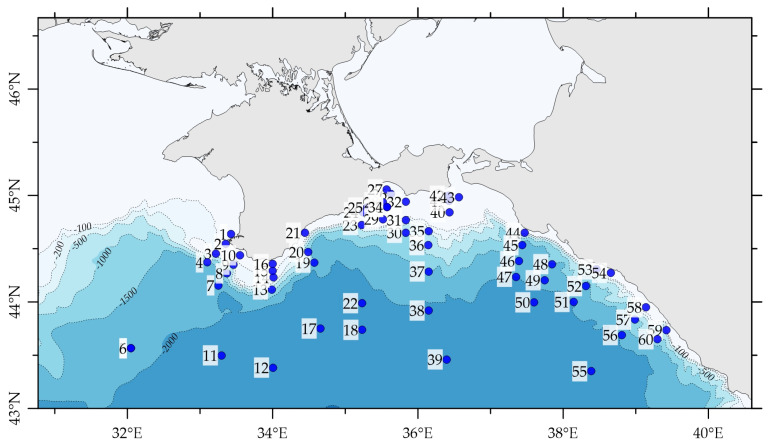
Sampling map for ^226^Ra and ^228^Ra activity definition during the 116 RV Professor Vodyanitsky cruise (22 April–17 May 2021).

The scheme of the sampling map is shown in Figure 1.

### 2.4. Determination of the Activity of ^226^Ra and ^228^Ra

The activity of radium isotopes was determined by two methods: gamma spectrometry and alpha–beta radiometry with radiochemical preparation according to the procedure described in [29].

For gamma–spectrometric determination of radium isotope activity, after drying, granular sorbents were placed in Petri dishes and sealed. When using fiber sorbents, the dried sorbent was ashed in a SNOL-30/1300-I1p muffle furnace (AB UMEGA-GROUP, Utena, Lithuania) at 700 °C for 8 h, then ash was placed in Petri dishes and sealed.

Three weeks after sealing (to achieve equilibrium between ^226^Ra and ^214^Pb), the activity of radium isotopes was measured on a CANBERRA multichannel gamma spectrometer for measuring X-ray and gamma radiation (Canberra Industries, Meriden, CT, USA) with a BE3825 detection unit for at least 24 h. The activity of ^226^Ra was determined by the daughter radionuclide ^214^Pb with an energy of 351.9 keV (q_γ_ = 37.2%), and ^228^Ra by the daughter ^228^Ac (T_1/2_ = 6.1 h, q_γ_ = 27.7%) with an energy of 911.6 keV. The gamma spectrometer was calibrated using a certified source of ^226^Ra, the geometry and density of which were identical to the measured samples.

The alpha–beta radiometry method for determining the activity of radium isotopes with radiochemical preparation according to the method in [29] can be used for sorbents based on manganese dioxide impregnated on a cellulose or fibrous support (DMM or PAN-MnO_2_). To eliminate the effect of short-lived radium isotopes, the sorbent with radionuclides adsorbed on it was aged for 2 weeks. In the coastal laboratory, the samples were leached with 2M HCl and hydroxylamine (ReaChem JSC, Moscow, Russia) to quantitatively remove the long-lived Ra isotopes. Then, Ra was coprecipitated with BaSO_4_ (ReaChem JSC, Moscow, Russia). The precipitate (100 mg) was transferred onto a mount. The counting sample prepared in this way then was aged for 4–5 days in a Petri dish and measured on an alpha–beta radiometer UMF-2000 (LLC “Scientific and Production Enterprise “Doza”, Zelenograd, Russia) for at least 8 h several times, the counting sample was measured again after 10–12 days.

The sorption efficiency of radionuclide from seawater was determined as [7]:

(1) $$E=1-\frac{B}{A},$$

where *A* and *B* are the activities of the radionuclide on the sorbent in the first and second adsorbers.

### 2.5. Main Biogenic Elements’ Content Determination

Together with sampling for radionuclides, samples for nutrient concentration were taken. DIP and silicic acid, as well as the sum of nitrates and nitrites in the surface layer and deep-sea waters of the Black Sea, were analyzed at the coastal laboratory in frozen samples.

The main elements of the main biogenic cycle were determined photometrically [30]: mineral phosphorus by molybdenum blue, and silicon by the blue silicomolybdenum complex. Nitrates (reduced to nitrites) and nitrites were determined by the azo dye formed upon interaction with the Griess reagent on a two-channel autoanalyzer “AutoAnalyzer AA II” (Bran+Luebbe GmbH, Norderstedt, Germany) [31].

The relative error of determination was 1.5–2% for DIP (concentration range: 0.2–8 µmol L^−1^), 0.13–2% for silicic acid (concentration range: 1.1–18.8 μmol L^−1^), and 0.01–0.1% for nitrates and nitrites (concentration range: 0–1 μmol L^−1^). Calibration curves for the determination of nutrients were given in Figure S1 in Supplementary Materials.

## 3. Results and Discussion

### 3.1. Assessment of ^226^Ra and ^228^Ra Recovery Efficiency by Various Sorbents

During the 116 RV Professor Vodyanitsky cruise, a survey about the possibility and efficiency of extracting radium isotopes from seawater using various types of sorbents was conducted.

**Figure 2:**
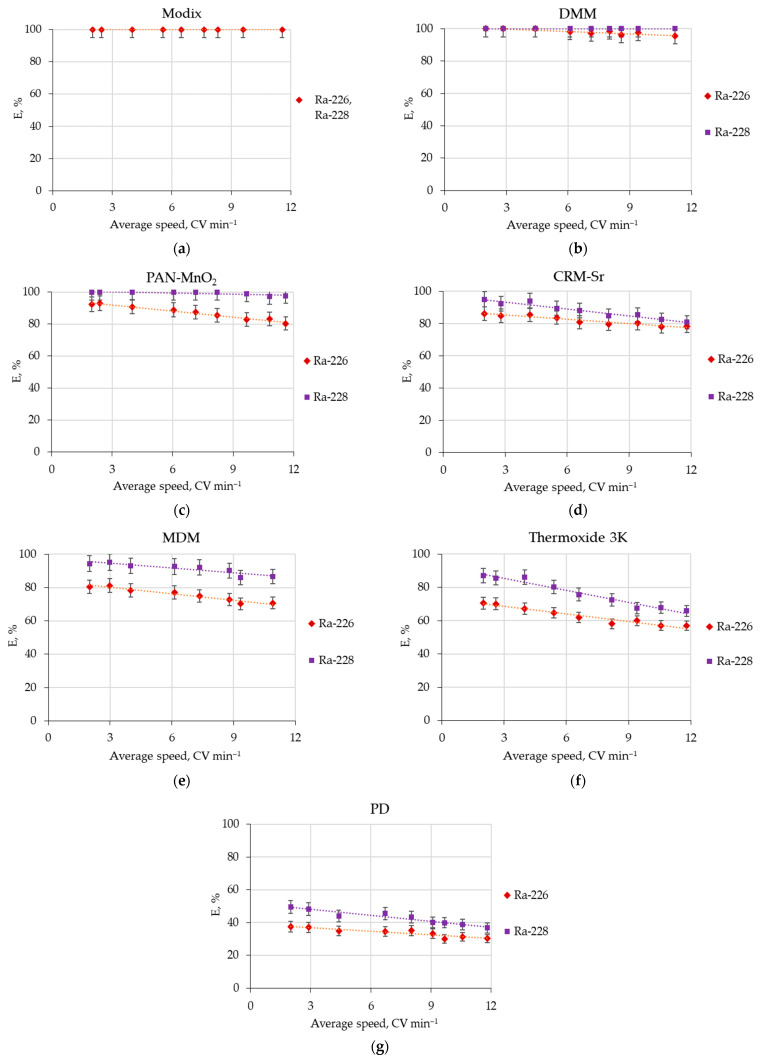
Dependence of ^226^Ra and ^228^Ra recovery efficiency (*E*, %) on the seawater flow rate by sorbents: (**a**) Modix; (**b**) DMM; (**c**) PAN-MnO_2_; (**d**) CRM-Sr; (**e**) MDM; (**f**) Termoxid 3K; (**g**) PD (two-column method, two columns with sorbents volume 25 mL each, seawater volume—250 L).

The results of the determination of the influence of seawater flow rate on the radium isotope recovery efficiency by various sorbents are shown in Figure 2.

The optimal seawater flow rate for the studied sorbents is 4–8 CV min^−1^. It is clear that the sorption efficiency for radium isotopes is more than 80% for the Modix, DMM, PAN-MnO_2_, and CRM-Sr sorbents and more than 60% for the MDM and Termoxid 3K sorbents.

Working under expeditionary conditions, to reduce the analysis time, an important technical task is to achieve a high seawater filtration rate through a fixed sorbent layer. In these conditions, the appropriate properties of the MDM sorbent with relatively large granules are shown. The use of highly dispersed sorbents such as Modix makes it difficult to achieve high filtration rates. Fibers impregnated with manganese dioxide (PAN-MnO_2_) have low hydrodynamic resistance and have proven themselves suitable for the sorption of radionuclides in expeditionary studies [27].

Radium sorption mechanisms are as follows:

- Sorbents based on manganese oxide (PAN-MnO_2_, Modix, MDM, DMM) [8]:

4Ra^2+^ + 6K_1.33_Mn_8_O_16_ → 8K^+^ + 6Ra_0.66_Mn_8_O_16_,

- Sorbents based on barium silicate (CRM-Sr) [32]:

Ra^2+^ + BaSiO_3_ → Ba^2+^ + RaSiO_3_,

- Sorbents based on carbonate-containing zirconium dioxide (Termoxid 3K) [33,34]:

2≡Zr-CO_3_H + Ra^2+^ + 2H_2_O → (≡Zr-CO_3_)_2_Ra + 2H_3_O^+^,

2≡Zr-CO_3_Na + Ra^2+^ → (≡Zr-CO_3_)_2_Ra + 2Na^+^,

- Sorbents based on carbonate-containing phosphorus oxide (PD) [27]:

2Ra^2+^ + (HO)_2_PO-O-*cellulosic support* → 2H^+^ + RaO_2_PO-O-*cellulosic support.*

**Figure 3:**
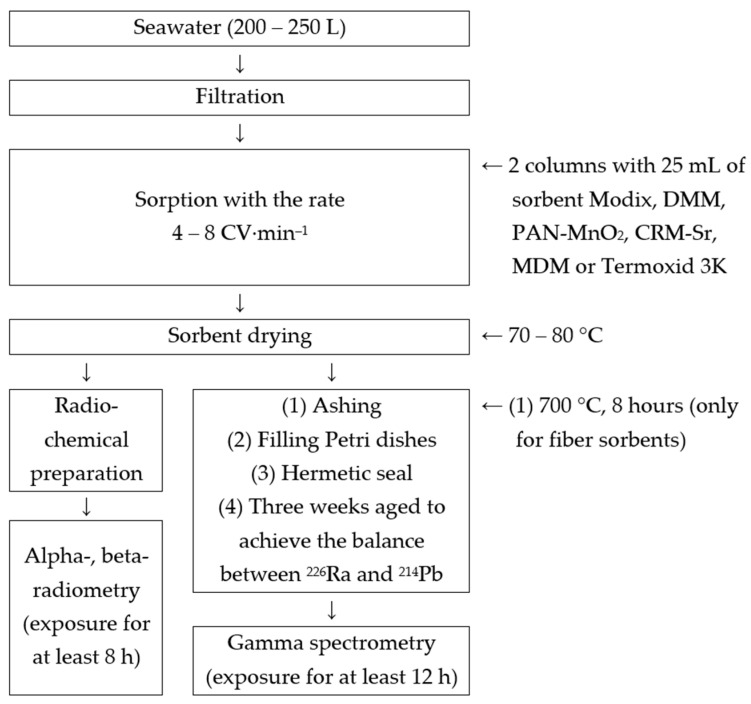
Scheme of sequence of steps taken in the method of ^226^Ra and ^228^Ra sorption.

Based on the results obtained, a procedure for extracting ^226^Ra and ^228^Ra from seawater was developed using our own and commercially available sorbents (Figure 3):

1. A 200–250 L volume of seawater is pumped into a container on the vessel board, simultaneously filtering seawater through a polypropylene filter with a pore diameter of 0.5–1 μm.
2. Two columns are filled with 25 mL of the Modix, DMM, PAN-MnO_2_, or CRM-Sr sorbent.
3. A 200–250 L volume of seawater is transmitted through columns with sorbents at a rate of 4–8 CV min^−1^.
4. After sorption, the sorbent is dried in an oven at a temperature of 70–80 °C.
5. When using granular sorbents, after drying, the sorbent is placed in Petri dishes and sealed. When using the PAN-MnO_2_ fibrous sorbent, the sorbent after drying is ashed in a muffle furnace at 700 °C for 8 h. The ash is placed in Petri dishes and sealed.
6. Measurement of the activity of ^226^Ra and ^228^Ra is carried out 3 weeks after sealing (to achieve equilibrium between ^226^Ra and ^214^Pb) on a semiconductor gamma spectrometer with an exposure of at least 12 h to achieve a measurement error of not more than 10%.
7. The sorption efficiency of ^226^Ra and ^228^Ra is calculated by Equation (1) for two absorbers.

It should also be noticed that for sorbents based on manganese dioxide impregnated on a cellulose or fibrous support (DMM or PAN-MnO_2_), it is possible to determine the activity of ^226^Ra and ^228^Ra by the alpha–beta radiometric method by washing manganese dioxide together with recovered radionuclides and further radiochemical preparation according to the method [29] described above.

This method combines the use of the most efficient sorbents with an optimal seawater flow rate, the simplicity of work in expeditionary conditions, the possibility of using equipment available for most laboratories, and the relative simplicity of sample preparation.

### 3.2. Distribution of ^226^Ra and ^228^Ra Radionuclides in the Surface Layer of the Black Sea in Spring 2021

**Figure 4:**
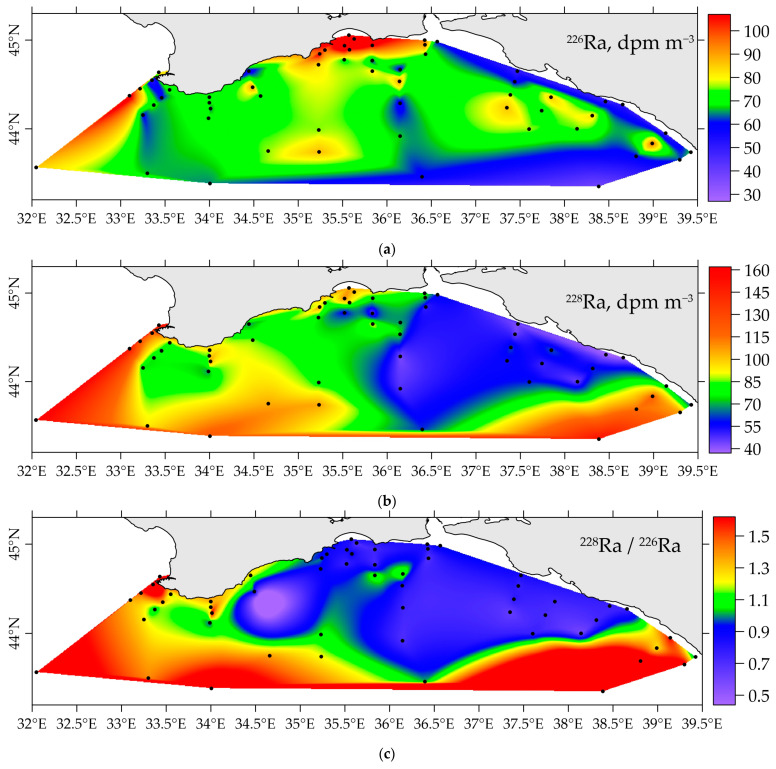
Distribution of ^226^Ra (**a**), ^228^Ra (**b**) concentration and the ratio of ^228^Ra/^226^Ra (**c**) in the surface layer of the Black Sea in April–May 2021.

**Figure 5:**
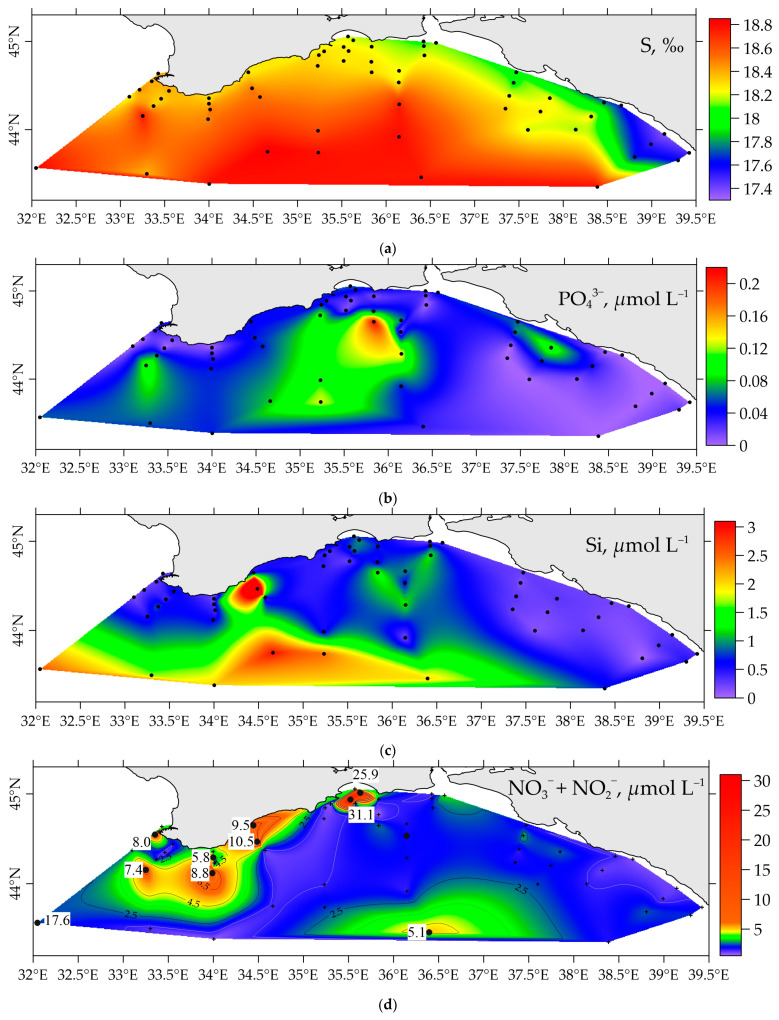
Distribution of salinity (**a**), dissolved inorganic phosphorus (**b**), silica (**c**), and the sum of nitrates and nitrites (**d**) in the surface layer of the Black Sea in April–May 2021.

In April–May 2021, 60 samples were taken to determine the concentration of radium isotopes in the surface layer of the Black Sea. It was shown (Figure 4) that increased concentrations of ^228^Ra are observed in the central part of the Black Sea, lowered near the coast of the Caucasus, which, in our opinion, is associated with lower salinity values and the input of fresh water with a higher content of ^226^Ra. The average concentration of ^228^Ra is 73 dpm m^−3^. The distribution of ^226^Ra is generally homogeneous and is in good agreement with the literature data. The average concentration is 77.2 dpm m^−3^ The highest concentrations were determined at four stations in Feodosia Bay, with values of 128–150 dpm m^−3^ for ^226^Ra and 100.2–110.5 dpm m^−3^ for ^228^Ra. At the same time, no lowered salinity was observed (Figure 5a), except for increased concentrations of nutrients (Figure 5b–d). In prospect, we plan to study this region in more detail to determine the source of radium in Feodosia Bay.

Using data on the ^228^Ra and ^226^Ra concentration and salinity, a quantitative assessment of the relationship between salinity and the concentration of these radionuclides was acquired. It is known [35,36,37] that all radium isotopes are supplied to the coastal waters in a ratio typical for each particular source. Owing to the short half-life, ^223^Ra (11.4 days) and ^224^Ra (3.66 days) are traditionally used to determine the mixing rate in the coastal region [38], while the long-lived ^226^Ra and ^228^Ra isotopes of radium are used to determine not only the water dynamics in the ocean [39,40] but also the type of freshwater coming with surface or underground runoff [36].

**Figure 6:**
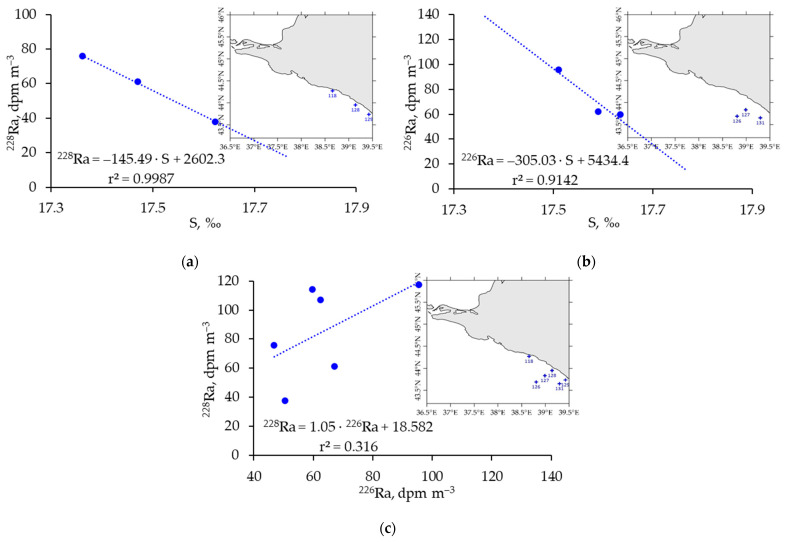
Dependence of ^228^Ra (**a**), ^226^Ra (**b**) concentration on salinity and the ratio of ^228^Ra/^226^Ra (**c**) for the least salty surface water region of the Caucasian coast (S < 18.0‰). The locations of seawater sampled stations are shown by crosses.

A detailed consideration of the relationship between long-lived radium isotope concentration and salinity led to the conclusion that at salinity S < 18.0‰, the highest correlation is observed at stations located along the Caucasian coast (Figure 6). There is significant freshening due to river runoff (Figure 5a). It is known that a significant amount of radium isotopes, both dissolved and adsorbed on the suspended matter, comes with river and groundwater discharge [41]. Desorption of Ra bounded to the particle surface occurs when particles meet high-ionic-strength water, i.e., freshwater mixes with seawater [42]. Conservative mixing of freshwater and seawater is the dominant process in the nearshore area, then with increasing distance from the coastline, the desorption processes continue. However, freshwater meets a great open seawater body, and the equilibrium of the long-lived Ra concentration cannot be reached instantly and is extended in time. Therefore, at stations located along the coastline at approximately the same distance from the coastline (Figure 6a), the best dependence of the ^228^Ra concentration on salinity was observed. Here, the process of conservative mixing of two end members, river and seawater, takes place [42]. The ^228^Ra/^226^Ra ratio for these stations was 0.183, which corresponds to the values in the SGD region of the island of Sicily in the Mediterranean Sea [43]. However, their ratio throughout the minimum values of the salinity area (Figure 5a and Figure 6c) was 1.05, which corresponds to the ratio of the ^228^Ra/^226^Ra activity available for desorption in the aquifer, which is close to the ^232^Th/^230^Th activity on the surface of solids (usually in the range of 0.5–1.5) [36].

With increasing distance from the coast, ^226^Ra highly correlates with salinity (Figure 6b). This is related to further processes of desorption of radium isotopes from the surface of suspended matter in river waters, which is supplied in large quantities in this region.

Thus, in this area (Figure 6c), which is most characteristic due to the significant river runoff, particularly in this observation period, owing to the difference in the half-lives of ^228^Ra (5.75 years) and ^226^Ra (1600 years) and due to the larger amount of ^226^Ra than ^228^Ra coming from river waters, equilibrium in the coastal zone is established faster for ^228^Ra than for ^226^Ra (Figure 6a,b).

The processes of conservative mixing of rivers and seawaters and further processes of radium desorption are shown by the distribution of long-lived radium isotopes and their ratio in the surface layer (Figure 4). Freshwater input can be traced not only in the coastal zone, but also in the far offshore part of the study area (Figure 4c). 

**Figure 7:**
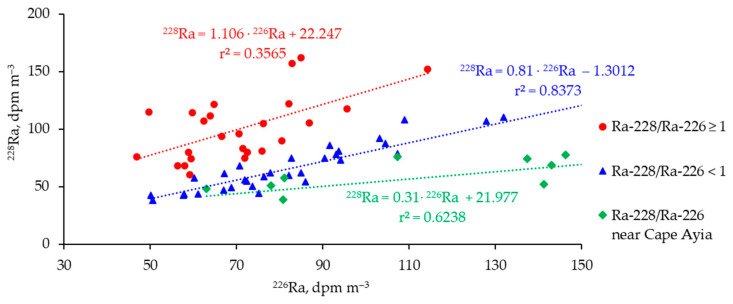
^228^Ra/^226^Ra ratio in the 116 cruise study area in comparison with the submarine groundwater source region near Cape Ayia.

In the vicinity area of the SGD source near Cape Ayia (southwest coast of Crimea, Black Sea) [44], the ratio of radium isotopes is significantly lower (Figure 7) (data obtained within the range from 0 to 100 m from the submarine groundwater source) than in the areas studied in the 116 RV Professor Vodyanitsky cruise (minimum offshore distance is within 4 km). Therefore, it can be said that at a great distance from the coastline, many processes affect the content of these radionuclides, such as the hydrodynamic processes of mixing, diffusion, and meandering of the Main Black Sea Current and, as a result, the involvement and transport of coastal waters into the abyssal areas of the sea. In other words, coastal waters are delivered to far offshore regions of the studied area, owing to various hydrodynamic processes.

### 3.3. Distribution of Nutrients in the Surface Water Layer of the Black Sea in Spring 2021

**Figure 8:**
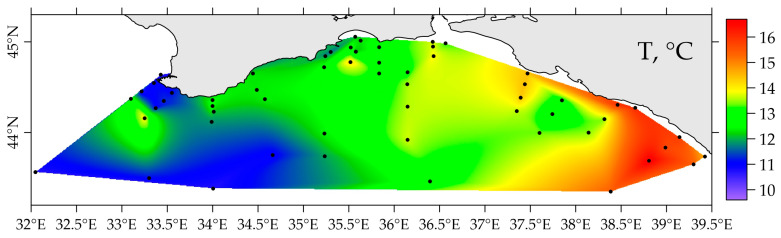
Distribution of temperature in the surface layer in spring 2021.

The spatial distribution of DIP and silicic acid, as well as nitrate and nitrite ions in the surface layer, is shown in Figure 5. The average DIP content in the surface layer of the region under consideration was 0.04 ± 0.04 (from 0.00 to 0.22 µmol L^−1^), and the content of silica was 0.7 ± 0.65 µmol L^−1^ (concentration range: 0.0 to 3.0 µmol L^−1^). The surface DIP distribution, silica, and nitrates in the period from 22 April to 17 May 2021, showed the lowest concentrations of these elements in the region of the Caucasian coast of the Black Sea (Figure 5b–d). Here, there is an increased temperature (Figure 8) and therefore elevated uptake of these elements by microplankton and lowered salinity (Figure 5a) due to river and underground discharge.

The minimum values of the sum of nitrate and nitrite ions were 0.53 µmol L^−1^, the maximum values were 31.13 µmol L^−1^, and the average values were 3.40 µmol L^−1^. The minimum concentrations were observed along the coast of the Caucasus, and the maximum in the area of Feodosia Bay, the Sevastopol region, and the southwestern coast of Crimea.

## 4. Conclusions

During the 116 RV Professor Vodyanitsky cruise, various types of sorbents based on manganese oxide (PAN-MnO_2_, Modix, MDM, DMM), phosphorus oxide (PD), carbonate-containing zirconium dioxide (Termoxid 3K), and barium silicate (CRM-Sr) were tested for the possibility and efficiency of extracting ^226^Ra and ^228^Ra from seawater.

It was defined that ^226^Ra and ^228^Ra sorption efficiency was more than 80% for the Modix, DMM, PAN-MnO^2^, and CRM-Sr sorbents and more than 60% for the MDM and Termoxid 3K sorbents at a seawater flow rate of 4–8 CV min^−1^. Based on the results obtained, a method for extracting ^226^Ra and ^228^Ra from seawater was developed.

The activities of ^228^Ra and ^226^Ra and their ratio obtained for the surface layer make it possible to trace the effect of freshwater runoff. The ratio of these isotopes is higher in the region of the surface runoff than that for the area of SGD (offshore distance within 100 m). This can be explained by the fact that with increasing distance from the coastline (from 4 to 48 km), the equilibrium is reached faster for ^228^Ra, owing to processes of desorption from riverine particulate matter and various long-lived radium half-lives. Therefore, a more detailed study of the water area adjacent to the submarine source will make it possible to study the behavior of these radionuclides in the Black Sea when they enter from SGD sources and thus identify the groundwater input in coastal regions.

The low content of phosphates and silica in the surface layer corresponds to elevated temperature and low salinity values in the corresponding area, which indicates the active involvement of these elements in biogeochemical processes. An increased value of nitrates and nitrites was observed in coastal areas, especially in Feodosia Bay (a slight increase in the concentration of silicic acid and rather high concentrations of ^226^Ra and ^228^Ra were also observed there). A more detailed study of Feodosia Bay is required to determine the source of radium in this region.

## Data Availability

Not applicable.

## Supplementary Materials

The following supporting information can be downloaded at: https://www.mdpi.com/article/10.3390/ma16051935/s1, Figure S1: Calibration curves for the determination of DIP, silicic acid, nitrates and nitrites.
