# Supplementary material for: Estimation of 226Ra and 228Ra Content Using Various Types of Sorbents and Their Distribution in the Surface Layer of the Black Sea
Source: Materials (Basel). 2023 Feb 26;16(5):1935. doi: 10.3390/ma16051935 (PMC10004327; doi:10.3390/ma16051935)
Supplement: Supplementary file 1 [file materials-16-01935-s001.zip › materials-2179223-supplementary.pdf]

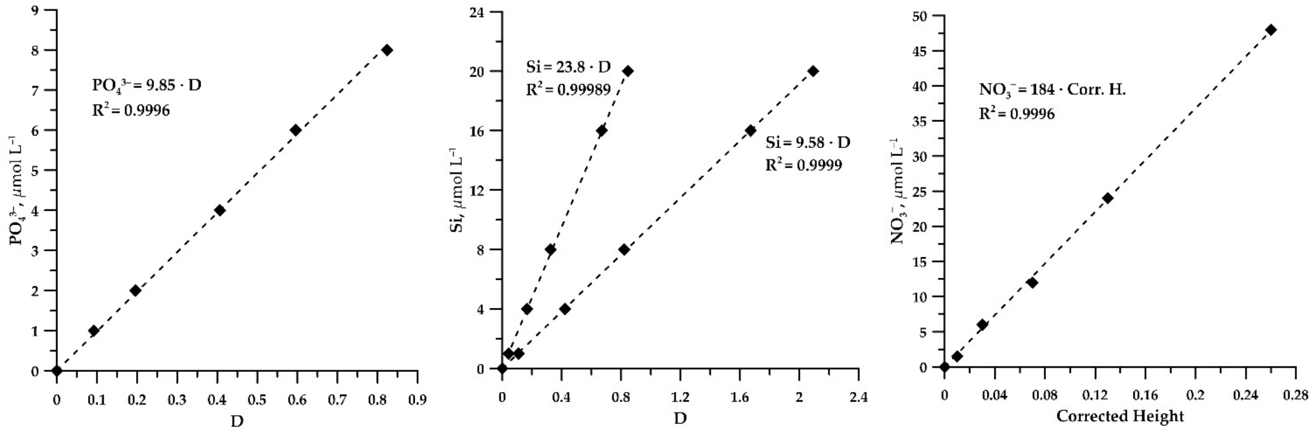

(**a**) (**b**) (**c**)

Figure S1. Calibration curves for the determination of DIP (**a**), silicic acid (**b**), nitrates and nitrites (**c**)
